# Effect of an Anaerobic Fermentation Process on 3D-Printed PLA Materials of a Biogas-Generating Reactor

**DOI:** 10.3390/ma15238571

**Published:** 2022-12-01

**Authors:** Adrian Cioabla, Virgil-Florin Duma, Corina Mnerie, Ralph-Alexandru Erdelyi, George Mihai Dobre, Adrian Bradu, Adrian Podoleanu

**Affiliations:** 1 Faculty of Mechanics, Polytechnic University of Timisoara, 1 Mihai Viteazu Ave., 300222 Timisoara, Romania; 2 3OM Optomechatronics Group, Faculty of Engineering, “Aurel Vlaicu” University of Arad, Str. Elena Dragoi No. 2, 310177 Arad, Romania; 3 Doctoral School, Polytechnic University of Timisoara, 1 Mihai Viteazu Ave., 300222 Timisoara, Romania; 4 Applied Optics Group, School of Physics, University of Kent, Canterbury CT2 7NR, UK

**Keywords:** bioengineering, anaerobic digestion, 3D-printed thermoplastic polymer, polylactic acid (PLA), surface topography, Optical Coherence Tomography (OCT), Scanning Electron Microscopy (SEM), Atomic Force Microscopy (AFM)

## Abstract

3D-printed materials are present in numerous applications, from medicine to engineering. The aim of this study is to assess their suitability for an application of interest today, that of testing of 3D-printed polylactic acid (PLA)-based reactors for biogas production using anaerobic digestion. The impact of temperature, pH, and aqueous phase on the tested bioreactor is investigated, together with the effect of the gaseous phase (i.e., produced biogas). Two batches of materials used separately, one after another inside the bioreactor were considered, in a realistic situation. Two essential parameters inside the reactor (i.e., pH and temperature) were continuously monitored during a time interval of 25 to 30 days for each of the two biogas-generating processes. To understand the impact of these processes on the walls of the bioreactor, samples of 3D-printed material were placed at three levels: at the top (i.e., outside the substrate), in the middle, and at the bottom of the bioreactor. The samples were analyzed using a non-destructive imaging method, Optical Coherence Tomography (OCT). An in-house developed swept-source (SS) OCT system, master–slave (MS) enhanced, operating at a central wavelength of 1310 nm was utilized. The 3D OCT images related to the degradation level of the material of the PLA samples were validated using Scanning Electron Microscopy (SEM). The differences between the impact of the substrate on samples situated at the three considered levels inside the reactor were determined and analyzed using their OCT B-scans (optical cross-section images). Thus, the impact of the biogas-generating process on the interior of the bioreactor was demonstrated and quantified, as well as the capability of OCT to perform such assessments. Therefore, future work may target OCT for in situ investigations of such bioreactors.

## 1. Introduction

Biofuels are currently a topic of interest because of their potential energetic input. They allow for obtaining partial autonomy in using locally produced residual sources of biodegradable materials for obtaining a clean energy carrier, as well as for the recovery of untapped sources for partially solving the increasing energy demand.

Biogas, as a renewable energy carrier, can be a possible solution to the present natural gas issues worldwide due to the multiple sources that can be used for its production and because it has as a main component methane, which is already used in both industrial and household fields of human activity. In this respect, anaerobic digestion or fermentation is the most common way to produce biogas in enclosed bioreactors. The process can take place in the absence of air (i.e., anaerobic fermentation), with different residence time for the tested substrate or with co-fermentation of multiple substrates such as animal waste, as well as agricultural or municipal biodegradable residual materials. It can be produced at different possible temperature regimes inside the test bioreactors. This process commonly develops four stages: hydrolysis, acidogenesis, acetogenesis, and methanogenesis [1,2]. During the first two phases, short-chained unsaturated products are obtained. They break down during acetogenesis and are transformed into acetic acid derivatives, carbon dioxide, and hydrogen [3]. In the last phase, methanogenic bacteria are produced by the degradation of fats and trace compounds. The final product consists of methane and carbon dioxide as main components [4]. Also, the resulting biogas can contain hydrogen sulfide, ammonia, water vapor, or siloxanes.

Co-digestion represents the process of anaerobic digestion for more than one substrate. It has a high impact on biogas yield and quality (i.e., it produces a high methane concentration by volume) [5,6]. In this regard, manure from animals (cow, swine, or poultry) is often used in different countries with positive results regarding biogas yield and quality [7,8]. Such a co-substrate that uses manure has a high buffer capacity to maintain a stable pH over time, which is a critical aspect for such processes [9].

Wastewaters represent another source of materials suitable for anaerobic digestion. They usually contain high concentrations of organic compounds and imply extensive costs regarding biological treatment and energy consumption [5,6,10,11,12]. Active sludge is an example directly related to wastewater processing. It relates to wastewater treatment plants (WWTP) that are necessary (and currently) used all over the world. Therefore, the residual material (i.e., wastewater and active sludge) can be easily found and used for anaerobic digestion processes. One cubic meter of wastewater from WWTP can contain between 3 and 6 MJ of potential heat energy according to the biological oxygen demand (BOD) and chemical oxygen demand (COD) concentrations [13].

However, activated sludge can be hazardous in different scenarios because of the contaminated waters which may enter the treatment plant. Pretreatment efficient processes are required and applied in this context [14]. Untreated activated sludge is hydrated to a level of 97–99%, while the rest is comprised of solid and dissolved matter, minerals and organic substances, coagulants, gels, and trapped gas bubbles. The stabilized sediment, on the other hand, is often hydrated only to a level of 60–88% [15,16,17].

Processed materials, both before and after anaerobic digestion, can be used as fertilizers in agriculture. One can perform an adaptation of the land to specific needs resulting from waste management activities, spatial development, compost production, or cultivation of flora not intended for public consumption [16]. This is done after an extensive analysis of the content of such materials in order to avoid potential contamination with heavy metals or other negative elements (e.g., dangerous microbes).

Related to bioreactors that should be built to accommodate such processes, current trends in materials engineering include the use of composites and polymers to obtain improved materials with appropriate characteristics. This belongs to a more general field of interest that includes automobiles, medical implants, electronics, aerospace, and robotics [18,19,20,21].

From a mechanical point of view, studies have determined that most thermoplastics present strong nonlinearities, viscous relaxations, strain rate dependences, thermal softening, and thermal transitions, because of the characteristics of their manufacturing processes [22,23,24,25]. In this respect, polylactic acid (PLA) is a thermoplastic polymer derived from renewable resources that contain rich carbohydrates, such as core and sugar cane [26,27,28]. Its applications include film, food packaging, textiles, as well as disposable bottles and tableware [26,27,29,30]. Several studies have found that natural fiber/PLA composites present higher mechanical properties compared to natural fiber/polypropylene (PP) or polyethylene (PE) composites, especially regarding their tensile modulus [31,32,33].

Considering all aspects above, the aims of the present study are:

- To assess PLA use for the development of a bioreactor for the testing of anaerobic digestion processes in a mesophilic temperature regime (i.e., 30 to 37 °C). The large variety of PLA applications and the fact that such materials are manufactured using renewable resources define the impact of this work.
- To determine if this material is suitable for such applications, considering the parameter impact and residence time of substrate, as well as of the produced biogas inside the vessel.
- To assess the capability of the main considered method to perform these investigations, Optical Coherence Tomography (OCT).

In the last three decades, OCT, an imaging method based on low coherence interferometry [34,35,36], has been extended to a wide area of biomedical applications that includes ophthalmology [37], dentistry [38,39,40], dermatology [41,42], and endoscopy [43,44]. Also, Non-Destructive Testing (NDT) for fields as diverse as industry [45,46,47,48,49] and art [50,51] has been approached.

In NDT, OCT has the potential for in situ investigations, with handheld scanning probes for easy access to different areas of interest of a sample [42,52,53,54]. Regarding the technique, OCT has evolved in terms of resolution and acquisition speed from early time domain (TD) to Fourier domain (FD) and swept-source (SS) OCT [35,36]. The latter is employed in the present study. Validation of qualitative OCT results is carried out in this work using Scanning Electron Microscopy (SEM).

## 2. Materials and Methods

### 2.1. Biogas-Generating Reactor and Testing Plates

**Figure 1:**
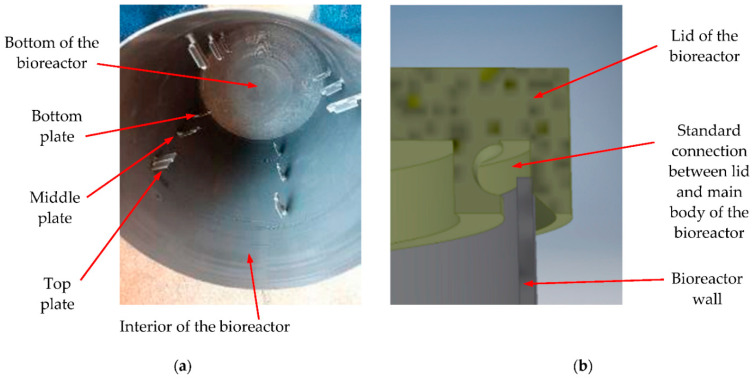
(**a**) Photo of the interior of the reactor before the beginning of the anaerobic process, with the three levels of testing plates; (**b**) connection between the vessel and the lid of the reactor.

An in-house developed bioreactor was employed in the study (Figure 1). A commercially available PLA material with a filament diameter of 1.7 mm produced by Shenzen Esun Industrial Co. (Shenzhen, China) was used to 3D-print this reactor. The fill factor utilized in the process was 100% to obtain the structure of the reactor as compact as possible with a low probability of micro-holes formation. Hence, the specific generated topography that is discussed in the following section.

The bioreactor was designed for a total volume of 6 L, from which approximately two-thirds was planned to be filled with the substrate suspension for a period of approximately 25 days.

**Figure 2:**
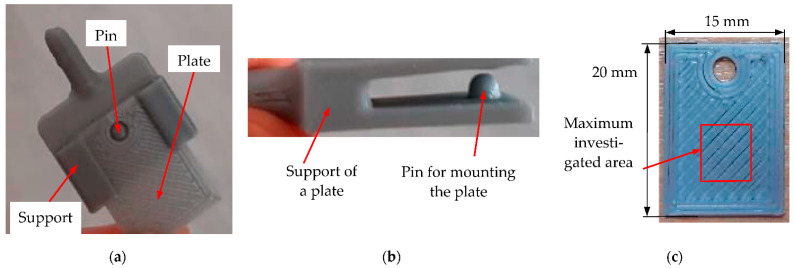
(**a**) Front view of a plate in its support before inserting in the bioreactor; (**b**) lateral view of a plate support, with its mounting pin; (**c**) photo of a plate utilized in the bioreactor (i.e., positioned at the top of the reactor, as shown in Figure 1a), indicating the dimensions of the twelve (identical) plates and the maximum (6 × 6 mm^2^) selected area for the different SEM and OCT investigations.

Figure 1 shows details of the bioreactor. The design of the standard connection between the lid and the bioreactor body (using their specific shape) is presented in Figure 1b. The sealing of the contained environment was secured using a silicone-based membrane. The general aspect of the interior of the reactor before the beginning of the biogas-generating process is shown in Figure 1a. Four testing plates were inserted in the reactor at three different levels: the lowest one, situated at 100 mm from the bottom of the reactor, the middle level in the mid-section, and the highest level at about 100 mm under the lid, the latter one in the zone that is not immersed in the liquid substrate. The design of a plate is shown in Figure 2. They were 3D-printed with the same technology as the bioreactor (therefore, they have the same surface topography), in order to be affected in the same way as the reactor wall by the biogas-generating process.

This distribution of the plates inside the reactor was planned in order to be able to observe the influence of the environment determined by the substrate at two levels of the liquid (i.e., near the bottom and in the middle section). The influence of the biphasic environment determined by the presence of biogas and vapors can be investigated using the plates positioned on the upper level. In order to make the investigations relevant, the plate’s material was identical to the material of the reactor walls. Each plate had a surface of 20 × 15 mm^2^ and a thickness of 2 mm—Figure 2a. The support of each plate (Figure 2b) was inserted in the body of the reactor (Figure 1a), and the plate was connected with the support via a small pin in order to stay fixed during the experiments (Figure 2).

The areas in which the plates were inserted had different levels of relative humidity, from 100% in the bottom and middle part of the bioreactor (i.e., where the liquid mixture was placed) to lower levels in the upper part, where there was a mixture of gas, vapors, and condensate. No visible influence that was due only to humidity could be spotted or reported.

After completing the testing, all plates were detached from the support and further analyzed in order to determine the effect of the biogas-generating process on the plate material, which is similar to the effect it had on the material of the reactor.

### 2.2. Biogas-Generating Process

According to the technical sheet for PLA, this material can stand unaffected by temperatures up to at least 60 °C. However, for a good anaerobic digestion process regarding the produced biogas versus the methane concentration and the obtained production, the options would be mesophilic process (with temperatures of 32 to 40 °C) or thermophilic process (with temperatures of 40 to 55 °C). The main difference between the two regimes consists in the residence time of the substrate inside the reactor. This time is reduced from periods of 20 to 30 days for a mesophilic process to periods of 1 to 3 days for a thermophilic process. However, for the latter process the quality of the produced biogas is greatly reduced in terms of methane concentration by volume. Also, the overall quantities of the produced biogas are larger for the mesophilic compared to the thermophilic regime. Hence, the first, lower temperature regime was chosen for the present study.

An experimental study was carried out with two consecutively used batches of material, each using as substrate a volume of 5 L of suspension. Each temperature-controlled bath (maintained at 37 to 38 °C) was inserted inside the reactor for a period of 30 days.

The monitoring process involved the measurement (and control) of both pH and temperature on a daily basis. Corrections of the pH, when necessary, were achieved using a 20% concentration solution of NH_3_. Temperature measurements were performed using a calibrated thermocouple and a digital thermometer, while pH measurements were completed with a Consort multiparameter analyzer (Consort bvba, Turnhout, Belgium).

The biogas produced during the process was measured using a gas analyzer model Biogas 5000 (Geotechnical Instruments Ltd., Warwickshire, UK), to quantify concentrations of methane, carbon dioxide, and hydrogen sulphide.

**Figure 3:**
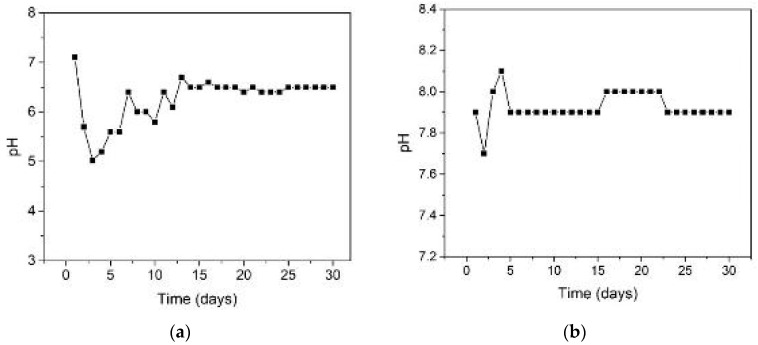
Time variation of the pH during the process for the first (**a**) and for the second batch used inside the bioreactor (**b**).

The first batch contains a mixture of 4.9 L of wastewater from a local treatment plant in Timis County, Romania, as well as 100 g of degraded corn grains. As it can be observed from Figure 3a, during the first 15 days of the process, the pH was corrected with the NH_3_ suspension, and the overall variation of the suspension pH was maintained between 5.5 and 6.5. After this first period, the pH reached a stable level in the neutral range specific to the process dynamics of 6.5 to 7. These values are normal for a good anaerobic digestion phenomenon.

The second batch has a more basic substrate, containing 5 L of a mixture composed of cow manure, corn silage, and chicken manure. Its pH value ranged from 7.6 to 8, which is in the upper range specific to this type of fermentation (Figure 3b). This is a good indicator of the high buffer capacity of the utilized substrate materials, as well as the high rate of biogas production over time.

In both scenarios, the reactors were planned to be filled to about two-thirds of their total volumes. To eliminate the air inside the vessels, initial washing with nitrogen was performed. Thus, after the formation of biogas, the upper part where the first level of plates was positioned presented a mixture of vapors and gas. The vapors were produced by the liquid suspension inserted in the reactor, because of the heat-exchanging phenomenon and the condensate formation on the internal part of the lid. The gas contains methane, carbon dioxide, and hydrogen sulphide as main components.

Both experiments produced biogas with an acceptable methane content (in the range of 50% to 60% concentration by volume). This proves that the type of material utilized for the bioreactor had no negative impact during the anaerobic fermentation process regarding the biogas production (i.e., it did not inhibit the formation of methanogenic bacteria). After carrying out both experiments in sequence, the plates were recovered, cleaned in a neutral solution, and naturally dried. They were consequently enclosed in sampling bags for further analyses.

### 2.3. Imaging Methods

OCT was carried out with an in-house developed swept-source (SS) OCT system, master–slave (MS) enhanced [55], equipped with a 50 kHz broadband laser source scanned in frequency (Exalos AG, Zürich, Switzerland), with a centre wavelength of 1310 nm. An in-house developed software (implemented in LabVIEW 2013, 64 bit) is used to acquire and process data. The digitization of the electrical signals at the output of the photodetector and driving the 2D galvanometer scanner was performed by two data acquisition boards, PXI5124 and PCI 6110 (National Instruments, Austin, TX, USA) [56]. The acquired channeled spectra are used to build a volumetric/3D OCT image by directly producing C-scans/en-face images (situated at a selected depth in the sample) using the MS protocol [55]. B-scans/optical cross-sections are obtained as well as utilized in this study. The system provides an axial resolution of 15 µm measured in air.

SEM was carried out with a high vacuum FEI Quanta 250 system (Thermo Scientific™ Quanta™, Hillsboro, OR, USA) equipped with a secondary Everhard–Thomley electron detector. Working parameters of the SEM, such as working pressure and distance, depended on the selected image. Each of the samples underwent the following investigating steps: mounting on a copper conductive holder stub with carbon wafers having adhesive on both sides; insertion and examination at an appropriate magnitude in the system; exposing of the investigated area directly to the scanning electron beam by mounting the sample guided by a binocular microscope. Each sample was aligned on the stub in order to eliminate its tilting inside this microscope. In order to obtain the best possible images, all investigated PLA samples were gold-coated.

## 3. Results

### OCT and SEM Images

**Figure 4:**
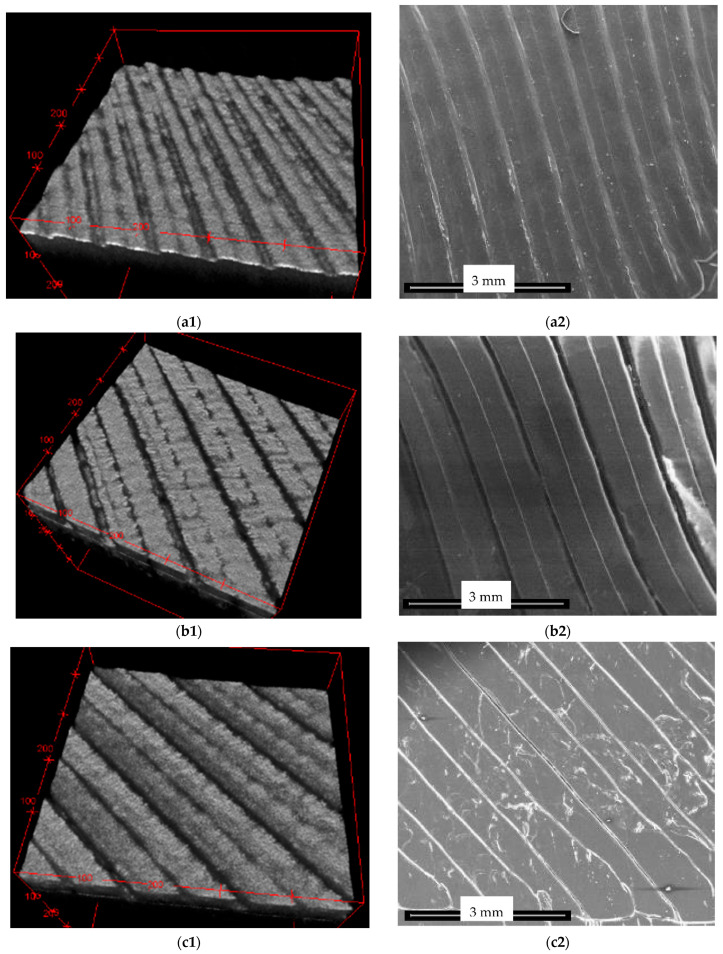

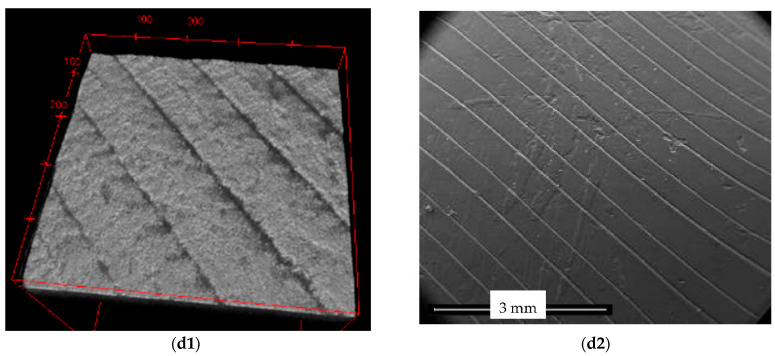
Imaging of the sample plates at different levels in the biogas reactor: (**a1**,**a2**) control plate; (**b1**,**b2**) top plate (situated in the upper part of the bioreactor); (**c1**,**c2**) middle plate (situated in the central part of the bioreactor); (**d1**,**d2**) bottom plate (situated in the densest part of the substrate in the bioreactor). Images in the column (**a1**–**d1**) on left represent 3D OCT reconstructions with a surface area of 3.5 × 3.5 mm^2^, while column (**a2**–**d2**) on right represent SEM images of the corresponding plate on left (gold-coated).

3D reconstructions of the OCT images of one of the four tested plates for each level inside the bioreactor are presented in Figure 4 in parallel with the validations performed using SEM.

One can observe the progressive degradation of the surface of the plates that are situated lower inside the bioreactor (i.e., from top to bottom) in comparison to the control plate, which is shown in Figure 4(a1,a2). Thus, the specific topography of the plates created by 3D printing (as pointed out in the beginning of Section 2), presented in Figure 4(a1,a2), shows only slight flattening for plates placed in the upper part of the reactor (i.e., in contact with gas and vapors)—Figure 4(b1,b2).

This surface topography suffers more flattening further on for plates in the mid-part of the reactor (Figure 4(c1,c2)), while plates at the bottom are almost completely flat. This phenomenon that can be observed qualitatively on 3D OCT reconstructions can be quantified using OCT B-scans, as carried on in the following.

Another aspect that can be concluded from Figure 4, this time by comparing the left and the right columns, is the good validation obtained for OCT volumetric reconstructions (left column) with higher-resolution SEM images (right column). Thus, one can conclude that OCT images (with 15 μm axial resolution, achievable by most OCT systems) can serve the scope of characterizing the impact of the reactor content on its walls. In this way, OCT may be considered for such evaluations instead of the more expensive and time-consuming SEM (even if its resolution is several orders of magnitude better, 4 nm).

**Figure 5:**
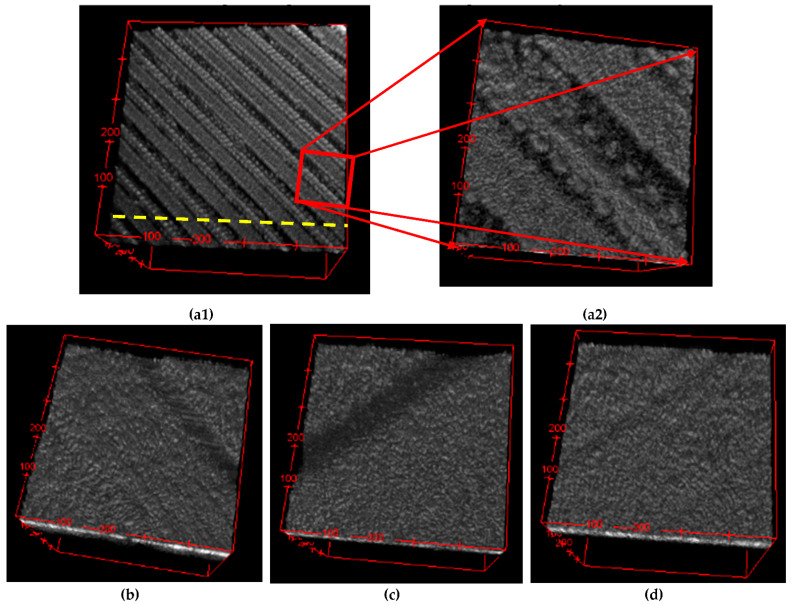
Details of 3D OCT reconstructions of sample plates situated in the biogas reactor: (**a1**) control plate with a surface area of 3.5 × 3.5 mm^2^—with a dashed line indicating the direction of the optical cross-sections/B-scans that are presented in the following figures; (**a2**) detail of a portion of the control plate with a surface area of 0.7 × 0.7 mm^2^, selected to show the surface topography; (**b**) detail of a top plate; (**c**) detail of a middle plate; (**d**) detail of a bottom plate. All images in the second row were selected in a similar way to the one in Figure 5a; they correspond to a surface area of 0.7 × 0.7 mm^2^.

Details from the 3D OCT reconstructions are presented in Figure 5, on areas that are about 25 times smaller than the areas in the images in Figure 4 and Figure 5(a1,a2). From the latter figure, the selected (0.7 × 0.7 mm^2^) area for imaging a detail of the profile of a control plate is marked. The same observation as above can be made regarding the flattening of plate surfaces that are situated deeper inside the reactor—from the complexity of the 3D-printed profile in Figure 5(a1,a2) to the almost nonexistent topography in Figure 5d.

**Figure 6:**
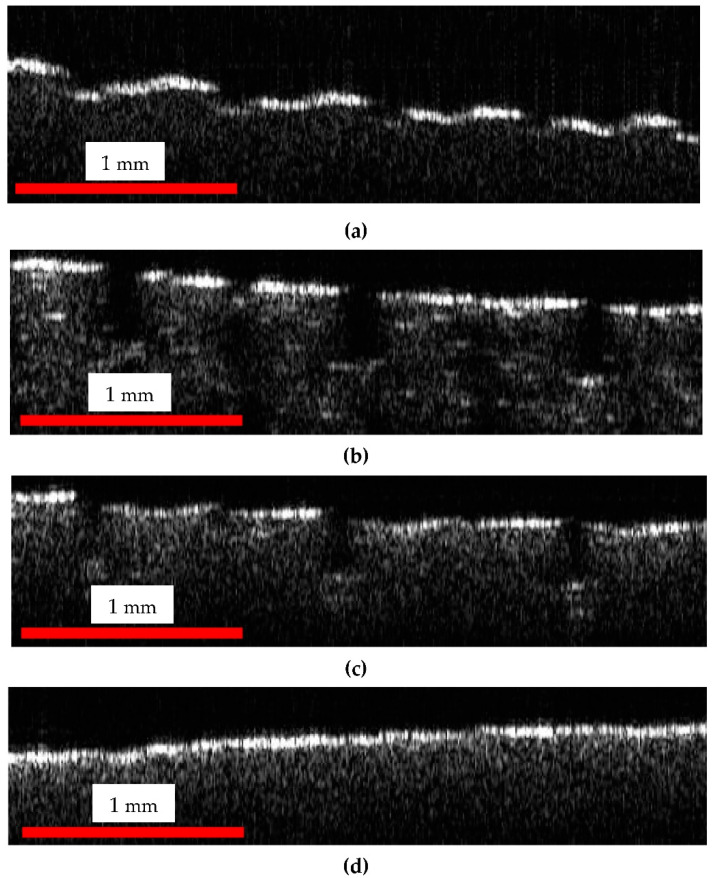
B-scans of sample plates from (**a**) the control group, (**b**) top, (**c**) middle, and (**d**) bottom zone of the bioreactor. The dimensions of the image are 3.5 (lateral) × 1.1 (vertical, along depth measured in air) mm^2^. B-scans are obtained parallel to the dashed line in Figure 5.

In order to quantitively characterize the phenomenon, Figure 6 presents an OCT B-scan (i.e., the 100th from the 500 performed B-scans) from the central part of a plate from each of the three levels within the bioreactor (as shown in Figure 1). Such B-scans/optical cross-sections are extracted from the 3D OCT images in Figure 4(a1–d1); therefore, they have a length of 3.5 mm.

**Figure 7:**
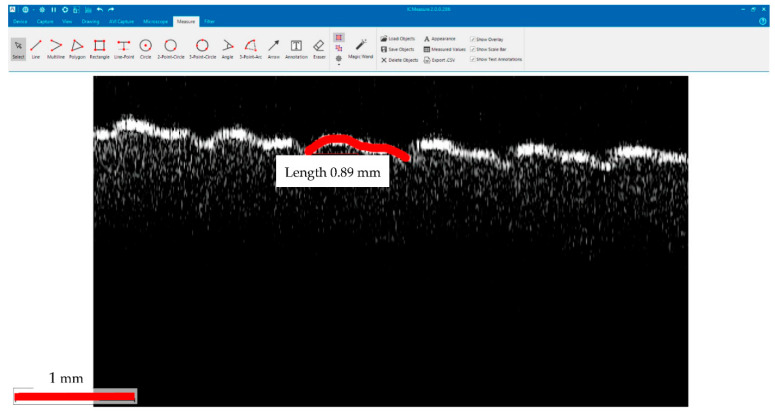
B-scan processed using the IC Measure program (The Imaging Source Europe GmbH, Bremen, Germany) in order to evaluate the lengths of the upper portions of each considered B-scan. The dimensions of the image are 3.5 (lateral) × 1.96 (vertical, along depth measured in air) mm^2^.

**Figure 8:**
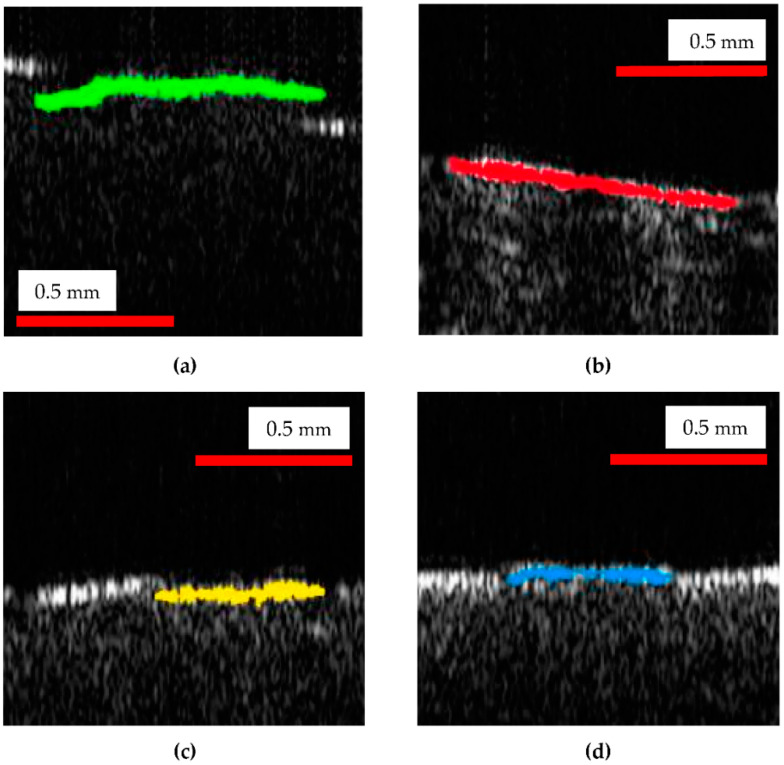
Details of OCT B-scans from each of the sample plates from: (**a**) the control group; (**b**) top plate; (**c**) middle plate; (**d**) bottom plate. The dimensions of each image are 0.92 (lateral) × 0.95 (vertical, along depth measured in air) mm^2^. The lengths in green, red, yellow and blue correspond to the length along the upper contour of the B-scans of each group between two consecutive gaps, as observed in Figure 7.

The differences in the surface topography of the four considered samples (and similar, in all four samples positioned at the same level inside the bioreactor) can be clearly noticed on all the images in Figure 4, Figure 5 and Figure 6. The parameter chosen to quantify these differences is the length of the upper portions on each of the four types of B-scans shown in Figure 6. Such lengths are measured on a curved contour, from gap to gap (as it is specific to the 3D-printed material, Figure 5(a1,a2)), as shown in Figure 7. They are further on compared, as pointed out by the colored portions in Figure 8.

The program used to process OCT B-scans in order to measure the lengths of the upper portions of the contours of the sample of B-scans, such as those in Figure 6 and Figure 7 (and marked with colors in Figure 8), is the free IC Measure (The Imaging Source Europe GmbH, Bremen, Germany). Such a program is specific for measuring details of 2D images (e.g., lengths or areas) imported after image calibration. These lengths were evaluated directly on B-scans, after the calibration of each image (Table 1). 

Fifty OCT B-scans were considered for measurements from each sample. They were chosen from the 500 B-scans used for each of the 3D reconstruction in Figure 4 (i.e., one from every patch of ten B-scans). Each of the six (upper) portions that can be seen in Figure 7 was measured for each B-scan; therefore, a total of three hundred values were evaluated for each of the four samples. For simplicity, only twenty values from each sample were provided in Table 1, as the mean and the standard deviation are very close to those calculated with all three hundred values.

From Table 1, one can observe that, compared to the reference, all the plates inserted in the suspension suffered dimensional modifications. In this regard, the top plates presented the least dimensional modifications, mainly because there was only a vapors/gas mix in the upper part of the reactor. This mixture had a reduced impact on the plates. In this area, the strongest impact can be attributed to the hydrogen sulphate present in the biogas, which can have a corrosive impact on the polymer-based surface.

The middle region presented a larger effect regarding dimensional reduction, due to the liquid phase that was surrounding the plates. The initial pH (Figure 3), which proved to be in the acid domain can be the main factor to influence the overall aspect and dimensional parameters of the plates.

The bottom plate presented an even greater reduced mean value for the considered dimensional aspect with regard to the presence of the combination of solid and liquid phase. Because of the gravitational factor, the solid part created a deposit on the bottom of the reactor. This aspect and the higher initial acidic values of the pH (i.e., less than 6), influenced the overall impact producing a further material dimensional reduction.

However, the presence (and thus, impact) of the (acid) liquid phase in both the middle and the bottom regions of the reactor is more likely the cause of the close values of the mean for the measured lengths for plates placed in these two regions.

The above quantitative results are in good agreement with the qualitative analysis made on the OCT and SEM images in Figure 4, Figure 5 and Figure 6.

Significant differences were obtained with the Kruskal–Wallis K statistics between the four groups, with a *p* < 0.00001 (while the result is significant at *p* < 0.01). Therefore, the medians of two or more groups are different.

By comparing the groups in pairs (Mann–Whitney U Test), the following results were obtained: (i) significant differences for the reference versus top values, as well as for reference versus middle or bottom measured values (*p* = 0.00001, while the result is significant at *p* < 0.01); (ii) significant differences for the top versus middle, as well as versus bottom measured values (with the same values and threshold of *p*); (iii) insignificant differences for the middle versus bottom values (*p* = 0.0536, while the result is significant at *p* < 0.01, as well).

## 4. Discussion

The biodegradable part of the different municipal sources of residual material (including wastewater) can be of interest regarding its potential use in anaerobic fermentation or co-fermentation processes. Such processes have as main result the production of biogas, which is a clean biofuel and can be further used in firing processes.

Different possibilities are presented in the literature for anaerobic digestion (or fermentation) for municipal residual materials [57,58], waste sludge and wastewaters from treatment plants [59,60,61] or from other industrial sources (such as bread or beer industry), glycerol-containing waters from the production of biodiesel [62,63], as well as residual materials from agriculture or households (i.e., degraded materials or dung from different sources) [64]. Therefore, the most important aspect is the untapped potential of those sources that are currently available in large areas in Romania and in the world.

Conventionally, bioreactors for this type of application are manufactured at small and medium scales from steel-based materials. Large-scale reactors dedicated to industrial processes are concrete-based enclosed spaces where the mixture of biodegradable substances is inserted for the biogas production process to occur.

The overall application for polymer-based materials is described in the literature [1,2,3,65,66,67], but there is little to no information about potential applications of those materials in anaerobic fermentation processes. In this context, the present study aimed to open a new direction in material utilization for biodegradation applications, as it involved the presence of PLA-based materials for bioreactors. The SEM and OCT analyses performed after the biodegradation experiments determined the overall impact of the parameters (such as temperature, pH, and biogas composition) on the bioreactor, in a way that was evaluated qualitatively with both methods, but also quantitatively using OCT.

This initial study can be a starting point for studying different polymer-based applications in anaerobic digestion processes. Thus, we can consider for future studies other materials of the reactor, as well as different parameters of substrates, this latter aspect referring to chemical components, local relative humidity, and heavy metals content.

The results of the quantitative assessment presented in Table 1 (and analyzed statistically in the previous section) shows the shrinking of the upper portions marked in colors in Figure 8 (extracted from the B-scans in Figure 6). The decrease of such lengths for the top plates inserted in the reactor with regard to control plates, as observed from the values of the means (for the evaluated lengths) in Table 1, is significant. Thus, the effect of the substrate placed inside the reactor on its PLA walls was demonstrated, even for top plates that are in contact only with vapors, not with the substrate itself. An even further decrease of these lengths can be observed from the top to the middle plates. Interesting, the values of the middle and bottom plates are close because of the strong impact of the liquid phase (and of its acidity) in both regions.

**Figure 9:**
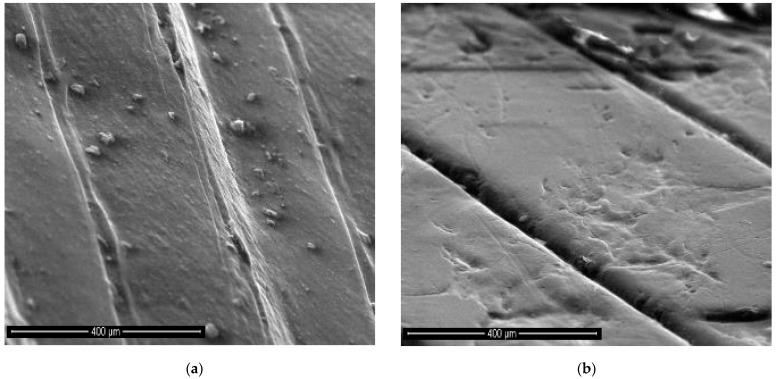
SEM image of (**a**) a control sample (that was not in contact with the biogas) versus (**b**) a middle sample—to highlight the level of degradation due to biogas exposure.

These quantitative results are in good agreement with the qualitative comparison based on 3D OCT images, as it was performed using Figure 4, Figure 5 and Figure 6. This comparison can also be made based on the SEM images in Figure 4. However, the 3D OCT images allow for a better assessment than the 2D SEM images, despite the much higher resolution of the latter. Thus, the impact of the substrate (with its pH and temperature) is clear from the flattening of the middle and bottom profiles of the 3D-printed PLA of the plates exposed to this substrate. This degradation of the PLA material (with surface flattening, but also with holes produced by the chemical exposure) can be seen very clearly in the (more) detailed SEM images in Figure 9.

One can conclude that the size of the different type of images is an issue: it must be carefully chosen to obtain the most clarity using each imaging technique. For OCT, this discussion can be made by comparing the larger images in Figure 4 to the details in Figure 5.

While OCT cannot provide details such as those in Figure 9 because of the major difference between the resolution of the two considered imaging methods (i.e., the micrometer range axial resolution for OCT versus the nanometer range resolution for SEM), OCT can perform the proposed assessment, not only qualitatively like SEM, but also quantitatively (based on B-scans), as demonstrated in the present study. Thus, as SEM can be much better in highlighting details (as observed in Figure 9), 3D OCT images are better at pointing out the evolution (i.e., the flattening) of the overall profile of the surface topography of the PLA samples with the depth of their position inside the reactor, as pointed out above. The former aspect is due to the much higher resolution of SEM, while the latter aspect is due to the fact that SEM images are 2D, while 3D OCT images allow for processing and extracting relevant information from optical cross-sections/B-scans, for example. The analysis performed in Figure 6, Figure 7 and Figure 8 and the data in Table 1, with the statistics that followed, show the capability of OCT to complete the proposed assessment on the impact of the reactor content on the material of its walls.

This material degradation may impact the lifetime of such bioreactors; therefore, future work in our groups are planned to address the durability of bioreactors, as well as their monitoring in time.

The present study shows that OCT can perform assessments of the integrity of bioreactors, at least in between batches of substrate (i.e., every 30 days). The study suggests the utility of perfecting the technology to allow for assessments in situ on working bioreactors using mobile OCT units equipped with handheld scanning probes [42,52,53,54].

The capability of SEM to validate OCT results was also pointed out and utilized in this work, as we have used in previous studies on metallic materials [48,49]. As a difference to OCT investigation that does not require any manipulation or processing of the sample imaged, for good SEM images the PLA samples are required to be metal (in this case, gold) coated. This aspect corroborated with the higher cost and more difficult operation of SEM systems compared to OCT ones is an argument in the OCT favor. Another argument in this respect refers to OCT’s capability to operate in situ and to target specific areas of interest of investigated structures using handheld probes, as pointed out above.

While, to our knowledge, this study is the first on the structural integrity of such bioreactors and on using OCT for their NDT, the complexity and possible high societal impact of the topic imposes further studies, including different substrates, biogas-generating processes, as well as other types, materials, and dimensions of the bioreactors.

## 5. Conclusions

The present study demonstrates that polymer-type materials such as PLA can be utilized for developing components and/or bioreactors at small scale for studying the anaerobic digestion process to produce biogas. Our experiments show that the testing plates utilized in the investigations are partially influenced by the chemical and biological reactions which take place inside the reactor. The developed structure allows for multiple testing and proves that this type of material is a possible solution to manufacture components for this type of applications. Although further research is needed on the chemical composition parameters and on different types of substrates used for testing, the findings in the present study are already promising for the potential use of PLA-based 3D-printed materials in this field of research.

Additionally, the study proves OCT’s capability to perform materials assessment for the bioreactor walls, at least in between batches introduced for biogas production. SEM can be a valuable method to validate OCT results, but it is not strictly required. Thus, the study demonstrates that OCT alone can perform both qualitative and quantitative assessments, while SEM can provide interesting supplementary data on details regarding the degradation of the walls. The differences between the impact of the vapors (in the upper part of the reactor) and of the substrate (in the middle and bottom part) on the bioreactor walls was demonstrated, as well.

This study can potentially open several avenues of research on: (i) PLA use for small scale bioreactors, which must continue for different reactor materials and a variety of substrates; (ii) OCT use for monitoring the impact of (different) substrates on bioreactors, eventually in situ, with mobile units and handheld probes; (iii) validation of OCT results with other imaging and testing methods, to determine the type of information each method can provide and the synergy that can be created between them.

## Figures and Tables

**Table 1:** Values of the lengths of the upper portions of the surface profiles of twenty B-scans, shown as examples marked with different colors in Figure 8, extracted from B-scans such as those shown in Figure 6. Evaluations using the program are shown on an example in Figure 7.

Sample	Reference (mm)	Top (mm)	Middle (mm)	Bottom (mm)
Measured values of the colored contours marked in Figure 8	0.91	0.82	0.59	0.56
	1	0.7	0.64	0.6
	0.81	0.65	0.56	0.61
	0.84	0.69	0.53	0.49
	0.88	0.86	0.61	0.62
	0.98	0.84	0.63	0.59
	0.91	0.8	0.57	0.52
	0.83	0.84	0.63	0.54
	0.9	0.83	0.68	0.58
	0.89	0.78	0.59	0.57
	0.86	0.81	0.55	0.61
	0.92	0.69	0.59	0.5
	0.84	0.85	0.65	0.62
	0.81	0.76	0.61	0.58
	0.95	0.74	0.57	0.61
	0.87	0.81	0.6	0.53
	0.98	0.73	0.59	0.57
	1	0.8	0.62	0.55
	0.93	0.79	0.58	0.59
	0.89	0.76	0.61	0.6
**Mean (mm)** $\bar{\varepsilon }=\frac{\sum_{1}^{N}\varepsilon_{j}}{N}$	**0.9**	**0.78**	**0.6**	**0.57**
Mean Absolute Deviation (mm) $MAD=\frac{\sum \left|x_{i}-\bar{x}\right|}{N}$	0.048	0.05	0.028	0.031
**Standard Deviation (mm)** $\sigma =\sqrt{\frac{\sum_{1}^{N}{\left(\varepsilon_{j}-\bar{\varepsilon }\right)}^{2}}{N-1}}$	0.059	0.059	0.036	0.038
Standard Error of the Mean (mm) $SEM=\sigma /\sqrt{n}$	0.0130	0.0131	0.0078	0.0085

## Data Availability

Data are available on request from the first author for biogas-generating processes and from the corresponding author for imaging and analyses.
